# Full blood count values as a predictor of poor outcome of pneumonia among HIV-infected patients

**DOI:** 10.1186/s12879-018-3090-0

**Published:** 2018-04-19

**Authors:** S. Camon, C. Quiros, N. Saubi, A. Moreno, M. A. Marcos, Y. Eto, S. Rofael, E. Monclus, J. Brown, T. D. McHugh, J. Mallolas, R. Perello

**Affiliations:** 1 Servicio de Urgencias, Hospital Clínic, Barcelona, Spain; 2 Servicio de Enfermedades Infecciosas, Hospital Clínic, Barcelona, Spain; 3 Servicio de Microbiología, Hospital Clínic, Barcelona, Spain; 4 Microbiology department, UCL, Royal Free Hospital, London, UK; 5 Pneumology department, Royal Free Hospital, London, UK

## Abstract

**Background:**

To evaluate the predictive value of analytical markers of full blood count that can be assessed in the emergency department for HIV infected patients, with community-acquired pneumonia (CAP).

**Methods:**

Prospective 3-year study including all HIV-infected patients that went to our emergency department with respiratory clinical infection, more than 24-h earlier they were diagnosed with CAP and required admission. We assessed the different values of the first blood count performed on the patient as follows; total white blood cells (WBC), neutrophils, lymphocytes (LYM), basophils, eosinophils (EOS), red blood cells (RBC), hemoglobin, hematocrit, mean corpuscular volume, mean corpuscular hemoglobin concentration, mean corpuscular hemoglobin, red blood cell distribution width (RDW), platelets (PLT), mean platelet volume, and platelet distribution width (PDW). The primary outcome measure was 30-day mortality and the secondary, admission to an intensive care unit (ICU). The predictive power of the variables was determined by statistical calculation.

**Results:**

One hundred sixty HIV-infected patients with pneumonia were identified. The mean age was 42 (11) years, 99 (62%) were male, 79 (49%) had ART. The main route of HIV transmission was through parenteral administration of drugs. Streptococcus pneumonia was the most frequently identified etiologic agent of CAP The univariate analysis showed that the values of PLT (*p* < 0.009), EOS (*p* < 0.033), RDW (*p* < 0.033) and PDW (*p* < 0.09) were predictor of mortality, but after the logistic regression analysis, no variable was shown as an independent predictor of mortality. On the other hand, higher RDW (OR = 1.2, 95% CI 1.1-1.4, *p* = 0.013) and a lower number of LYM (OR 2.2, 95% CI 1.1-2.2; *p* = 0.035) were revealed as independent predictors of admission to ICU.

**Conclusion:**

Red blood cell distribution and lymphocytes were the most useful predictors of disease severity identifying HIV infected patients with CAP who required ICU admission.

**Electronic supplementary material:**

The online version of this article (10.1186/s12879-018-3090-0) contains supplementary material, which is available to authorized users.

## Background

Community-acquired pneumonia (CAP) is the most common infection affecting HIV infected patients presenting to the Emergency Department (ED) [1], and one of the major causes of death due to infectious aetiology, as well as in the general population [2]. Among the HIV infected patients, the highly active antiretroviral therapy (ART) has the most consistent effect on reducing pneumonia and in the patients with fewer than CD4 count 200 cells / μl, antimicrobial prophylaxis is usually effective [3]. The identification or recognition of biomarkers rapidly obtained together with clinical scores and guidelines [4, 5], having allowed us to predict the severity of the HIV infected patients with CAP, would help us to optimize treatment and its management. The blood count is a laboratory test, done to any patient who visits an Emergency Department (ED) with an infectious clinic of any aetiology. These results are obtained quickly.

A number of biochemical and haematological markers have been used to assess systemic inflammation or infection as pneumonia in the clinical setting, in the develop countries. Particularly C-reactive protein levels, erythrocyte sedimentation rate, white blood cell counts, haemoglobin levels, and procalcitonin levels [6], which have an important value in predicting outcomes in severe infections [7–9]. Most of the biomarker studies are performed in the general population whereas in the HIV-infected population, the studies done with biomarkers are generally not immediately available, such as interleukins [10], or in patients with opportunistic infections [11, 12]. Some of them, showed that some values or the full blood count, as haemoglobin and CD4 count, were predictors of poor outcome [13], but they do not make specific reference of CAP.

Therefore, our group evaluated the prognosis of HIV-infected patients diagnosed with CAP based on blood count values, since their results are obtained quickly and can be useful in the management of this pathology.

## Methods

### Patients

This is a prospective three-years (2011-2013) at a large university hospital caring for HIV-infected patients in Barcelona, Spain. It included all the HIV infected patients that attended our ED with respiratory clinical history of more than 24 h of evolution and were diagnosed with CAP and required admission. Patients with high suspicion of *P. jirovecii* and/or tuberculosis on the basis of clinical and radiological presentation, plasma lactate dehydrogenase levels and/or Ziehl sputum staining, those who had received antibiotic treatment during the last 7 days, required hospitalization within the previous 15 days and patients from other hospitals were not included in this study.

### Definitions

The CAP was defined according to the criteria of the Infectious Disease Society of America (IDSA) [14], that include radiographic criteria (pulmonary condensation, cough, fever, among others. It diagnostic was made at ED. The intensive care unit (ICU) admission criteria at our center are based on the IDSA / ATS recommendations [15]. Patient with severe immunosuppression was defined as having a CD4 count < 350 cells/μl.

### Variables

For the purpose of this study, the following epidemiological, clinical and laboratory variables were collected: gender, age, previous number of CD4 lymphocytes, and viral load (VL) prior to admission, treatment with ART, co-infection with hepatitis C virus. Values of the full blood count (no longer than 2 h after presentation to ED the blood test was drawn), such as total white blood cells (WBC), neutrophils, absolute lymphocytes (LYM), basophils, eosinophils (EOS), red blood cells (RBC), hemoglobin, hematocrit, mean corpuscular volume, mean corpuscular hemoglobin concentration, mean corpuscular hemoglobin, Red blood cell distribution width (RDW), platelets (PLT), mean platelet volume, and platelet distribution width (PDW) (the values of measure units and normality intervals in our center can be seen in Additional file 1), the score of APACHE II scale, systolic blood pressure, heart rate, respiratory rate and arterial oxygen pressure, and C-reactive protein on the arrival to our ED. In addition, admission to an ICU and 30-days mortality were analysed.

### Laboratory studies

In order to obtain a microbiological diagnosis, a nasopharyngeal swab to detect respiratory viruses was used (Viral Culturette, Direct, Becton-Dickinson Microbiology Systems, MD, USA), urinary antigens to identify *Streptococcus pneumoniae* (BinaxNOW *S. pneumoniae* Urinary Antigen Test, USA) and *Legionella pneumophila* (BinaxNOW Legionella Urinary Antigen Test, USA) in concentrated urine in advance, and two blood cultures (Bactec 9240; Becton Dickinson, USA) before administering the antibiotic were performed. A sputum sample was collected for Gram stain and culture when the patient could expectorate before administration of the antibiotic.

To assess the severity of the condition, the APACHE II score was used (as previously studies) [16] because FINE scale [17], which is regularly used, is not standardized to use for immunosuppressed population.

### End- points

The primary end-point was, that full blood counts values, can predict 30-day mortality, related with CAP. Patients were followed by telephone during 3 months.

The second end-point was that full blood counts values, can predict ICU admission during hospitalization related with CAP.

### Statistical analysis

As for the statistical calculations, the categorical variables were expressed as frequencies and percentages, and the continuous variables were expressed as mean and standard deviation. Factors associated with ICU admission and mortality were assessed by univariate and multivariate analyses. Results were considered statistically significant when P was less than 0.05. For independent samples, Student’s t-test was used to evaluate continuous variables and the chi-squared test was selected to evaluate categorical variables. All statistical analyses were calculated by SPSS version 20.0 (Chicago, IL, USA).

## Results

One hundred sixty HIV-infected patients diagnosed with CAP were included to this study. The median age (IQR) was 42 (11) years, 99 (62%) were males, 79 (49%) were under ART, 9 (6%) were unaware of their HIV infection and their diagnosis was held in the same ED. The median (IQR) total CD4 count was 240 (405) cells/μl, and the viral load in blood was 815 (40,331) RNA copies/mm^3^. There was HCV co-infection in 95 (60%) patients. The main route of HIV infection was the use of parenteral drugs. The remaining variables can be seen in Table 1.

**Table 1:** Clinical and analytical characteristics of the 160 patients included in the study

	Total cohort	No ICU	ICU
Age (IQR^a^)	42 (11)	43 (11)	41 (12)
Gender (male%)	99 (62%)	74 (67%)	25 (63%)
Total CD4 (cells/mm3) (median (IQR))	242 (406)	350 (102)	247 (359)
Viral load in blood (copies RNA/mm3) (median (IQR))	709 (39961)	702 (36631)	5200 (111000)
Undetectable viral load < 200 copies/mL (n (%)^b^)	62 (39%)	50	12
Parental drug abuse as a risk factor for HIV (n (%))	94 (59%)	24	70
ART (n (%))	78 (49%)	61 (51%)	18 (45%)
APACHE II Scale Score (mean (IQR))	14 (7)	10 (6)	16 (7)
Heart rate (median (IQR))	96 (24)	94 (31)	97 (22)
Respiratory rate (median(IQR))	22 (8)	20 (6)	26 (11)
Partial pressure of oxygen in blood (mmHg) (mean (IQR))	60 (16)	65 (20)	55 (12)
HCV Co-Infection (n (%))	94 (59%)	34	60
Blood leukocytes (cells/mm3) (mean (IQR))	9 (7)	9 (7)	12 (11)
C-reactive protein (mg/dl) (mean (IQR))	14 (21)	13 (18)	19 (23)

^a^IQR: interquartile range

^b^Undetectable viral load: HIV viral load in blodd < 200 copies/mL

A bacterial diagnosis was confirmed in 90 (60%) patients. The most commonly diagnosed bacterium was *S. pneumoniae* in 81 (51%) patients, and no microbiological diagnosis was obtained in 70 (49%). Blood culture, Gram stain and culture, urinary antigen were positive in 34 (21%), 32 (21%) and 52 (33%) patients for *S. pneumoniae*, respectively. The Gram stain was positive in 14 patients, while in the other 20, was the cultured necessary to get the diagnosis. Respiratory viruses were isolated in 14 patients, being rhinovirus as the most frequently isolated respiratory virus in 6 (4%) patients (Table 2).

**Table 2:** Microbiological identification

Respiratory pathogens.	N
Bacteria	
*Streptococcus pneumoniae*	81(51%)
*Legionella pneumophila*	2(1%)
*Haemophillus influenzae*	5(3%)
*Streptococcus pyogenes*	1(1%)
*Staphylococcus aureus*	1(1%)
Virus	
Rhinovirus	6(4%)
Adenovirus	5(3%)
Influenza A Virus	2(1%)
Influenza B Virus	1(1%)

Forty patients required admission to an ICU (25%), and 21 (13%) required mechanic ventilation. Patients who died were 9 (6%) patients, and all died in the ICU. In the nine patients who died during their hospital stay, *S. pneumoniae* was the bacterium detected in most occasions (6 cases), 1in the sputum culture, 3 in the urinary antigen and in 2 in the blood culture. In two patients, no microbiological diagnosis was acquired, and in one case, the rhinovirus was found as the only microorganism. The variables (Table 3), assessed in the univariate analysis were only the blood count, and showed that mortality group had significant lower values of PLT (112.7+/− 57.6 vs. 196.55+/− 102.6; *p* < 0.009) and EOS (0.044+/− 0.039 vs. 0.108+/− 0.138; *p* < 0.033), and higher values of RDW (17.2+/− 2.9 vs. 15.3+/− 2.4; *p* < 0.033) and PDW (20.6+/− 5.0 vs. 17.5+/− 10.7; *p* < 0.09). Direct logistic regression was performed to asses full blood counts variables that can predict mortality or ICU admission. The variables included in the model were PLT, EOS, LYM, PDW and RDW. No other variables were included in the model. The full models containing all predictors were statistically significant in both cases (mortality: chi-square = 13.4; *p* = 0.009; ICU: chi-square = 14.0; *p* = 0.016), however none of the analysed variables were evaluated as an independent predictor of mortality. On the other hand, a higher RDW (OR = 1.2, 95% CI 1.1-1.4; *p* = 0.013) and a lower number of LYM (OR 2.2, 95% CI 1.1-2.2; *p* = 0.035) were shown as independent predictors of ICU admission. The rest of the analysed variables were not predictive factors of poor outcome.

**Table 3:** Logistic regression

	Mortality		ICU Admission	
	OR (IC 95%)	P	OR (IC 95%)	P
PLT (10^9^/L)	0.99 (0.98 – 1.00)	0.069	1.00 (0.99 – 1.00)	0.933
EOS (%)	0.00 (0.00 – 41.22)	0.128	1.42 (0.06 – 35.49)	0.830
RDW (%)	1.30 (0.97 – 1.73)	0.080	1.21 (1.04 – 1.41)	0.013
PDW (%)	1.02 (0.95 – 1.09)	0.643	0.99 (0.96 – 1.04)	0.864
LYM (%)	1.37 (0.53 – 3.55)	0.516	0.45 (0.22 - 0.95	0.035

*PLT* Platelets, *EOS* Eosinophils, *RDW* Red blood cell distribution width, *PDW* Platelet distribution width, *LYM* Lymphocytes. Units: (10^9^cells/L)

There were no statistically significant differences among the patients who had a microbiological diagnosis, those who did not have any, and those who presented severe immunosuppression (*p* > 0.05). Patients with several immunosuppression diagnoses didn’t present significant mortality (*p* > 0.05), the CD4 counts it’s not statistically different between both groups in the univariate analysis so the CD4 counts not seems to be the reason for this relationship.

On the other hand, a higher RDW (OR = 1.2, 95% CI 1.1-1.4; *p* = 0.013) and a lower number of LYM (OR 2.2, 95% CI 1.1-2.2; *p* = 0.035) were shown as independent predictors of ICU admission.

## Discussion

We hypothesized that the different values of full blood count can predict pneumonia outcome in HIV-infected patients, but statistic analysis showed opposite results according the primary end-point. We studied the value of the blood count as it is rapidly and widely available at relatively. For its interpretation of results it must be taken into account that in the HIV-infected patient anemia, leukopenia, and thrombocytopenia are the most common hematological abnormalities resulting from HIV infection [18].

HIV infection is associated with chronic inflammation and an increased risk of thrombotic events so that platelet indices may offer a rapid and affordable method to monitor platelet activation and disease progression in patients with HIV [19, 20]. Platelet aggregation plays a crucial role in the immune defense mechanism against viruses and bacterial LPS and subsequently forming platelet leukocyte aggregate. It is known that platelet monocyte aggregation correlates with markers of immune activation, disease progression and platelet aggregation in HIV treatment [21]. But despite the important role they play in the entire infectious process, and in sepsis-associated coagulopathy [22], in our study neither the PLT value nor the PDW value showed a predictive value of poor prognosis.

As for the WBC value, our study utilized both the frequency of EOS and total LYM and compared count of EOS with previous studies. These results were in accordance with the same ones performed by our group previously [23], which showed that they were not a factor of poor prognosis, in terms of mortality. LYM can generate more discussion, since they depend on many variables and not always from HIV, and a high level of LYM does not means a better immune state. Bordon et al. [24] showed that total CD4, CV and ART variables were not a predictor of poor prognosis in HIV infected patients with CAP. These results coincide with our study, so we believe that our study still provides further evidence on this subject. LYM only showed predictors markers of ICU admission. We know that this affirmation could be a point of intensive discussion, because several studies showed on the contrary [25] maybe by the methodology used, but several meta-analysis must be done in this subject.

As the RBC, is known, that ART can alter many haematological figures. Therefore, diagnosis of thalassaemia, anisocytosis and anemia should be evaluated carefully in combination with those parameters [26]. Anemia is an important marker of uncontrolled HIV infection [27], and in non-HIV infected patients with pneumonia it is an independent predict risk factor [28].

One of the most important RBC markers is probably RWD, which remains a powerful marker of cardiovascular disease [29]. However, our study showed no predictive value for mortality like the study by Braun et al. [30], in which RDW elevation was associated with higher morbidity and mortality while it was performed in the general population. RDW was predictor as LYM of ICU admission.

Respiratory failure remains the highest cause of ICU admission in HIV infected patients, accounting for 25 to 40% of the admissions [31], neutrophil-to-lymphocyte ratio is a good predictor for ICU admission in elderly no-HIV adults with CAP [32].

Nowadays, using medicine in which high technology is widely established, the collection of vital signs in the same head of the patient, a good medical history and physical examination, combined with a calculation of a severity score and a series of laboratory values at an affordable cost, could be sufficient to predict the prognosis of CAP in HIV infected patients, and identify those that require more complex management, without relying on other more expensive tests.

### Limitations

Our study has been implemented in a single hospital center, although it was with an important sample to obtain conclusions. This study could have a serious bias since only those who required hospitalization were included. CAPs caused by opportunistic microorganisms have not been included. Moreover, pneumococcal and/or influenza vaccination variables have not been included, either. Another limitation was that HIV positive population mostly acquired HIV infection through IV drug use, and had relatively low levels of virological suppression and relatively low CD4 counts. Given this small number patients with the outcome, it would be difficult to detect a significant finding with multivariable analysis even if blood counts were truly associated with prognosis. The small number of outcomes was another limitation.

## Conclusion

In HIV infected patients with CAP**,** an increase in RDW and a decrease in the number of absolute LYM were shown to be independent predictors of ICU admission. Although no statistically significant associations were observed between blood counts and mortality, numbers were too small to draw firm conclusions.

## Additional files

Additional file 1: Standard blood count values. (DOCX 14 kb)

Additional file 2: Informed consent. (DOCX 10 kb)

## Abbreviations

ART: Highly active antiretroviral therapy
CAP: Community acquired pneumonia
ED: Emergency department
EOS: Eosinophils
ICU: Intensive care unit
IDSA: Infectious Disease Society of America
LYM: Lymphocytes
PDW: Platelet distribution width
PLT: Platelets
RBC: Red blood cells
VL: Viral load
WBC: White blood cells

## References

[1] Lee CC, Hsiech CC, Chan TY, Chen PL, Chi C, Ko WC (2015). Community-onset febrile illness in HIV-infected adults: variable pathogens in terms of CD4 counts and transmission routes. Am J Emerg Med.

[2] Camón S, Perelló R, Escoda O, Escoda R, Aguilar N, Saubi N (2014). Reason for HIV Patients Consultation to the Emergency Department in the HAART Era: Incidence and Mortality. J AIDS Clin.

[3] Feikin DR, Feldam C, Schuchat A, Janoff EN. Global strategies to prevente bacterial pneumonia in adults with HIV disease. Lancet infect Dis. 2004;5:445–56. 10.1016/S1473-3099(04)01060-6 15219555

[4] Viale P, Petrosillo N, Castelli F, Cadeo B, Giampiero C, for the POP-IHV Study Group (2002). Algortithm-based Management of pneumonia in HIV-1-infected patients. Lancet.

[5] Chew KW, Yen IH, Li JZ, Winston LG (2011). Predictors of pneumonia severity in HIV infected adults admitted an urban public hospital. AIDS Patients Care STDS.

[6] Michail M, Jude E, Liaskos C, Karamagiolis S, Makrilakis K, Dimitroulis D (2013). The performance of serum inflammatory markers for the diagnosis and follow-up of patients whith osteomyelitis. Int j low extreme wounds.

[7] Rodriguez-Fernandez A, Andaluz-Ojeda D, Almansa R, Justel M, Eiros JM, Ortiz de Lejarazu R (2013). Eosinophil as a protective cell in *S aureus* ventilador-associated pneumonia. Mediators Inflamm.

[8] Abidi K, Khoudri I, Belayachi J, Madani N, Zekraoui A, Zeggwagh AA (2008). Eosinopenia is a reliable marker of sepsis on admission to medical intensive care units. Crit Care.

[9] Liu H, Liu G, Tian Z (2014). Changes in blood lymphocytes in sepsis patients. Zhonghua Wei Zhong Bing Ji Jiu Yi Xue.

[10] Benito N, Moreno A, Filella X, Miró JM, González J, Pumarola T (2004). Inflammatory responses in blood of HIV-infected patients with pulmonary infections. Clin Diagn Lab Immunol.

[11] Grant PM, Komarow L, Sanchez A, Sattler FR, Asmuth DM, Pollard RB (2014). Clinical and immunologic predictors of death after an acute opportunistic infection: results from ACTG A5164. HIV Clin Trials.

[12] Watanabe H, Kobayashi S, Watanabe K, Oishi K, Sanchai T, Kositsakulchai W (2000). Pulmonary infection caused by Rhodococcus equi in HIV-infected patients: report of four patients from northern Thailand. J Infect Chemother.

[13] Duong T, Jourdain G, Ngo-Giang-Huong N, Le Cœur S, Kantipong P, Buranabanjasatean S (2012). Laboratory and clinical predictors of disease progression following initiation of combination therapy in HIV-infected adults in Thailand. PLoS One.

[14] Barttlett JG, Breiman RF, Mandell LA, File TM, JR. (1998). Community-acquired pneumonia in adults: guidelines for management. The Infectious Diseases Society of America. Clin Infect Dis.

[15] Nseir S, Grailles G, Soury-Lavergne A, Minacori F, Alves I, Durocher A (2010). Accuracy of American Thoracic Society/Infectious Diseases Society of America criteria in predicting infection or colonization with multidrug-resistant bacteria at intensive care unit admission. Clin Microbiol Infect.

[16] Afessa B, Green B (2000). Bacterial pneumonia in hospitalized patients with HIV infection: the pulmonary complications, ICU support, and prognostic factors of hospitalized patients with HIV(PIP) study. Chest.

[17] Fine MJ, Auble TE, Yealy DM, Hanusa BH, Weissfeld LA, Singer DE (1997). A prediction rule to identify low-risk patients with community-acquired pneumonia. N Engl J Med.

[18] Enawgaw B, Alem M, Addis Z, Melku M (2014). Determination of hematological and immunological parameters among HIV positive patients taking highly active antiretroviral treatment naïve in the antiretroviral therapy clinic of Gondar Universtuty hospital, Gondar, Northwest Ethiopia a comparative cross-secttional study. BMC Hematol.

[19] Nkambule BB, Davison G, Ipp H (2015). The evaluation of platelet indices and markers of inflammation, coagulation and disease progression in treatment-naïve, asyntomatic HIV-infected patients. Int J Lab Hematol.

[20] Nkambule BB, Davison G, Ipp H (2015). The value of flow cytometry in the measurement of platelet activation and aggregation in human immudodeficiency virus infection. Platelets.

[21] Nkambule BB, Davison G, Ipp H (2015). Platelet leukocyte aggregates and markers of platelet agrgregation, immune activation and disease progression in HIV- infected treatment naïve asymptomatic individuals. J Thromb Thrombolysis.

[22] Lyons PG, Micek ST, Hampton N, Kollef MH. Sepsis-Associated Coagulopathy Severity Predicts Hospital Mortality. Crit Care Med. 2018; Epub ahead of print 10.1097/CCM.0000000000002997 29373360

[23] Perelló R, Miró O, Miró JM, Moreno A (2008). Role of the eosinophil count in discriminating the severety of community-acquired pneumonia in HIV infected patients. Crit Care.

[24] Bordon J, Kapoor R, Martinez C, Portela D, Duvvuri P, Klochko A (2011). CD4+ cell counts and HIV-RNA levels do not predict outcomes of community acquired pneumonia in hospitalizaed HIV-infected patients. Int J Infect Dis.

[25] Lamas CC, Coelho LE, Grinsztejn BJ, Veloso VG (2017). Community-acquired lower respiratory tract infections in HIV-infected patients on antiretroviral therapy: predictors in a contemporary cohort study. Infection.

[26] Al-Kindi SG, Zidar DA, McComsey GA, Longenecker CT (2017). Association of anisocytosis with markers of immune activation and exhaustion in treated HIV. Pathog Immun.

[27] Miller V, Phillips AN, Clotet B, Mocroft A, Ledergerber B, Kirk O (2002). Association of viruses load CD4 cell count, and treatment with clinical progression in human immunodeficiency virus-infected patients with very low CD4 cell counts. J Infect Dis.

[28] Reade MC, Weissfeld L, Angus DC, Kellum JA, Milbrandt EB (2010). The prevalence of anemia and its association with 90-day mortality in hospitalized community-acquired pneumonia. BMC Pulm Med.

[29] Al-Kindi SG, Kim CH, Morris SR, Freeman ML, Funderburg NT, Rodriguez B (2017). Brief report: elevated red cell distribution width identifies elevated cardiovascular disease risk in patients with HIV infection. J Acquir Immune Defic Syndr.

[30] Braun E, Kheir J, Mashiach T, Naffa M, Azzam ZS (2014). Is elevated red cell distribution width a pronostic predictor in adult with community acquiered pneumonia?. BCM infec Dis.

[31] Van Lelyveld SF, Wind CM, Mudrikova T, Van Leeuwen HJ, de Lange DW, Al H (2011). Short-and long-termoutcome of HIV-infected patients admitted to the intensive care unit. Eur J Clin Microbiol Infect Dis.

[32] Cataudella E, Giraffa CM, Di Marca S, Pulvirenti A, Alaimo S, Pisano M (2017). Neutropil-To-Lymphocyte ratio: An emergencing marker predicting prognosis in elderly adults with community-acquired pneumonia. J Am Geriatr Soc.

