# Supplementary material for: Full blood count values as a predictor of poor outcome of pneumonia among HIV-infected patients
Source: BMC Infect Dis. 2018 Apr 19;18:189. doi: 10.1186/s12879-018-3090-0 (PMC5909258; doi:10.1186/s12879-018-3090-0)
Supplement: Supplementary file 1 — Standard blood count values. (DOCX 14 kb) [file 12879_2018_3090_MOESM1_ESM.docx]

Standard blood counts values. Hospital Clinic, Barcelona

| **Heamatological results** | **Result** | **Unit** |
| --- | --- | --- |
| Leukocytes | 7.42 | 10^9^/L |
| Hematies | 4.36 | 10^12^/L |
| Haemoglobin concentration | 127 | g/L |
| Hematocrit | 0.4 | L/L |
| Mean Cell Volume | 92.2 | fl |
| Mean Cell Hemoglobin | 29.1 | pg |
| Mean corpuscular hemoglobin concentration | 316 | g/L |
| Reed distribution Width | 14.0 | % |
| Platelet Count | 322 | 10^9^/L |
| Mean Platelet Volume | 9.2 | fl |
| Neutrophils | 60.9 | % |
| Lymphocytes | 29.7 | % |
| Monocytes | 5 | % |
| Eosinophils | 0.9 | % |
| Basophils | 1.8 | % |
| Neutrophils abs | 4.5 | 10^9^/L |
| Lymphocytes abs | 2.2 | 10^9^/L |
| Monocytes abs | 0.4 | 10^9^/L |
| Eosinophils abs | 0.1 | 10^9^/L |
| Basophils abs | 0.1 | 10^9^/L |
