# Supplementary material for: Full blood count values as a predictor of poor outcome of pneumonia among HIV-infected patients
Source: BMC Infect Dis. 2018 Apr 19;18:189. doi: 10.1186/s12879-018-3090-0 (PMC5909258; doi:10.1186/s12879-018-3090-0)
Supplement: Supplementary file 2 — Informed consent. (DOCX 10 kb) [file 12879_2018_3090_MOESM2_ESM.docx]

I am….. I consent to the inclusion of my data in a database that is only accessible to Dr. Rafael Perello for the study: FULL BLOOD COUNT VALUES AS A PREDICTIVE FACTOR OF POOR OUTCOME IN PNEUMONIA AMONG HIV-INFECTED PATIENTS.

I have been informed that the database is anonymous and strictly confidential, and that if I do not want to participate in the study, my health care will not be different from the habitually rendered. Also I can request the withdrawal of the data when I deem it appropriate. In no case if biological samples are stored for future studies

Sing up

Data

DNI (Identity card Spanish)

Main investigator

Rafael Perelló MD, PhD.
